# Cardiac organoid — a promising perspective of preclinical model

**DOI:** 10.1186/s13287-021-02340-7

**Published:** 2021-05-06

**Authors:** Dandan Zhao, Wei Lei, Shijun Hu

**Affiliations:** Department of Cardiovascular Surgery of the First Affiliated Hospital & Institute for Cardiovascular Science, Collaborative Innovation Center of Hematology, State Key Laboratory of Radiation Medicine and Protection, Medical College, Soochow University, Suzhou, 215000 China

**Keywords:** 3D culture, Cardiac organoid, Stem cells, Heart disease models

## Abstract

Human cardiac organoids (hCOs), three-dimensional (3D) cellular constructs similar to in vivo organ, are new-generation models. To a large extent, a hCO retains the biological characteristics and functions of cells in vivo more accurately than previous models. With the continuous development of biotechnology, the hCO model is becoming increasingly complex and mature. High-fidelity hCOs help us better explore the mysteries of human physiology and integrate phenotypes with living functions into models. Here, we discuss recent advances in the methods of constructing human cardiac organoids and introduce applications of hCOs, especially in modeling cardiovascular diseases, including myocardial infarction, heart failure, genetic cardiac diseases, and arrhythmia. In addition, we propose the prospects for and the limitations of hCOs. In conclusion, a greater understanding of hCOs will provide ways to improve hCO construction and make these models useful for future preclinical studies.

## Introduction

**Fig. 1:**
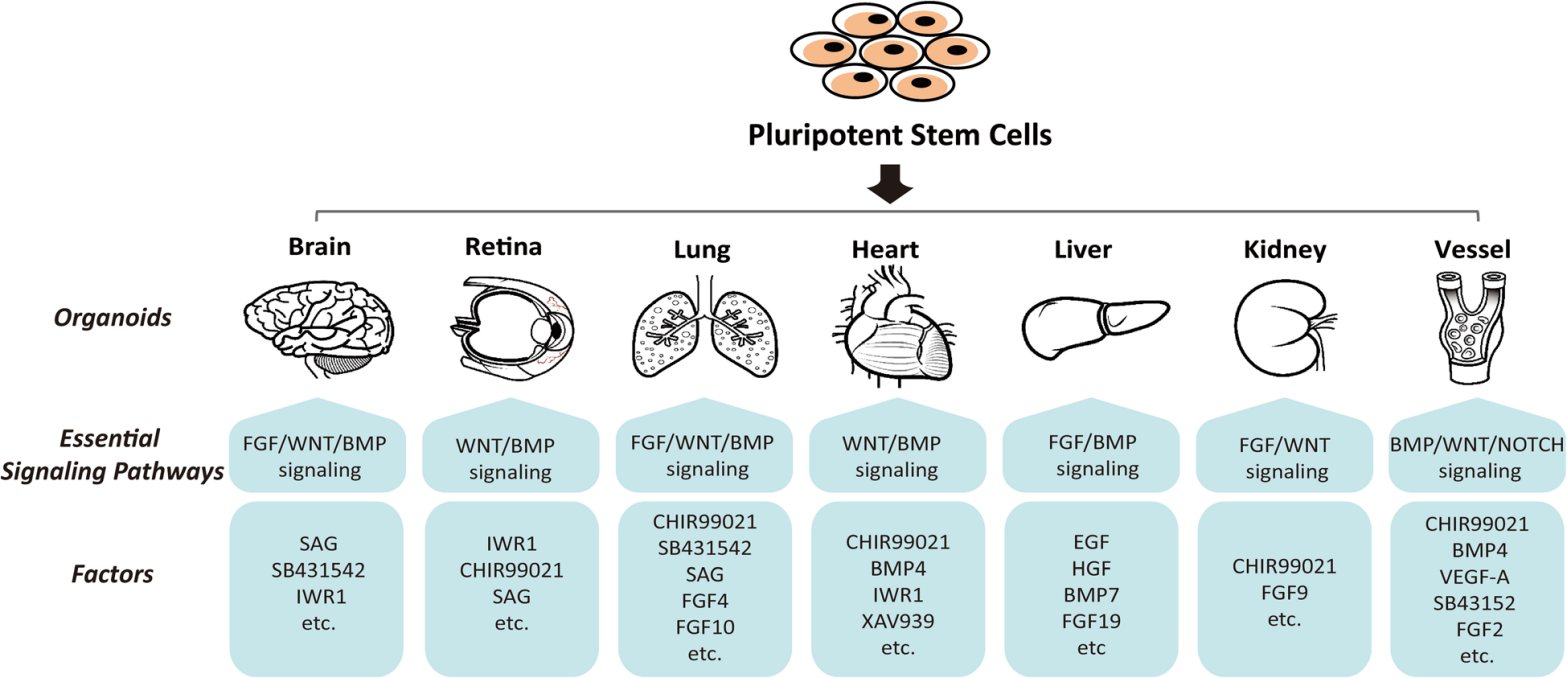
Essential signaling pathways and key factors for the construction of various organoids with pluripotent stem cells. FGF, fibroblast growth factor; BMP, bone morphogenetic protein; SAG, Hedgehog agonist smoothened agonist; SB431542, a TGFβ inhibitor; IWR1, a WNT inhibitor; CHIR99021, a GSK3β inhibitor; XAV939, a WNT inhibitor; EGF, epidermal growth factor; HGF, hepatocyte growth factor; VEGF, vascular endothelial growth factor

Organoid technology is one of the most popular and influential three-dimensional (3D) culture advancements in the field of biological and medical research. Scientists believe that organoids are organ-like multicellular constructs that are formed randomly or directionally by using the inherent self-assembly property of stem cells [1]. Organoids can mimic the key characteristics of their representative organs: (a) they contain more than one cell type similar to the original organs, (b) the organization of these cells is similar to that of organs in vivo, and (c) some specific functions of the original organs are recapitulated [2]. Most organoid construction protocols require specific exogenous signals because the original cellular system does not contain all the necessary components to undergo self-organization. In some cases, exogenous signals are needed only to induce the initial cell type, with the remaining self-organizing steps dependent on the autonomous signals in the system [3]. Specific factors are used to simulate the microenvironment in vivo to maintain cell survival and to self-assemble into organoid structures [4–6] (Fig. 1). For approximately a decade, scientists have reported on organoids of the brain [7], retina [8], lung [9], heart [10], liver [11], kidney [12], vessels [13], and other systems [14]. Organ-like constructs simulating physiological and pathophysiological development can be used to study various aspects of relevant organs in vitro [15, 16]. Especially in the face of global epidemics, organoids provide a valuable experimental platform for understanding the spatiotemporal effect of SARS-CoV-2 infection and pathogenesis of COVID-19 [17–20]. Overall, organoids contribute to a better understanding of the deeper problems associated with tissue development, homeostasis, diseases, and the integration of phenotypes with living function [21].

**Fig. 2:**
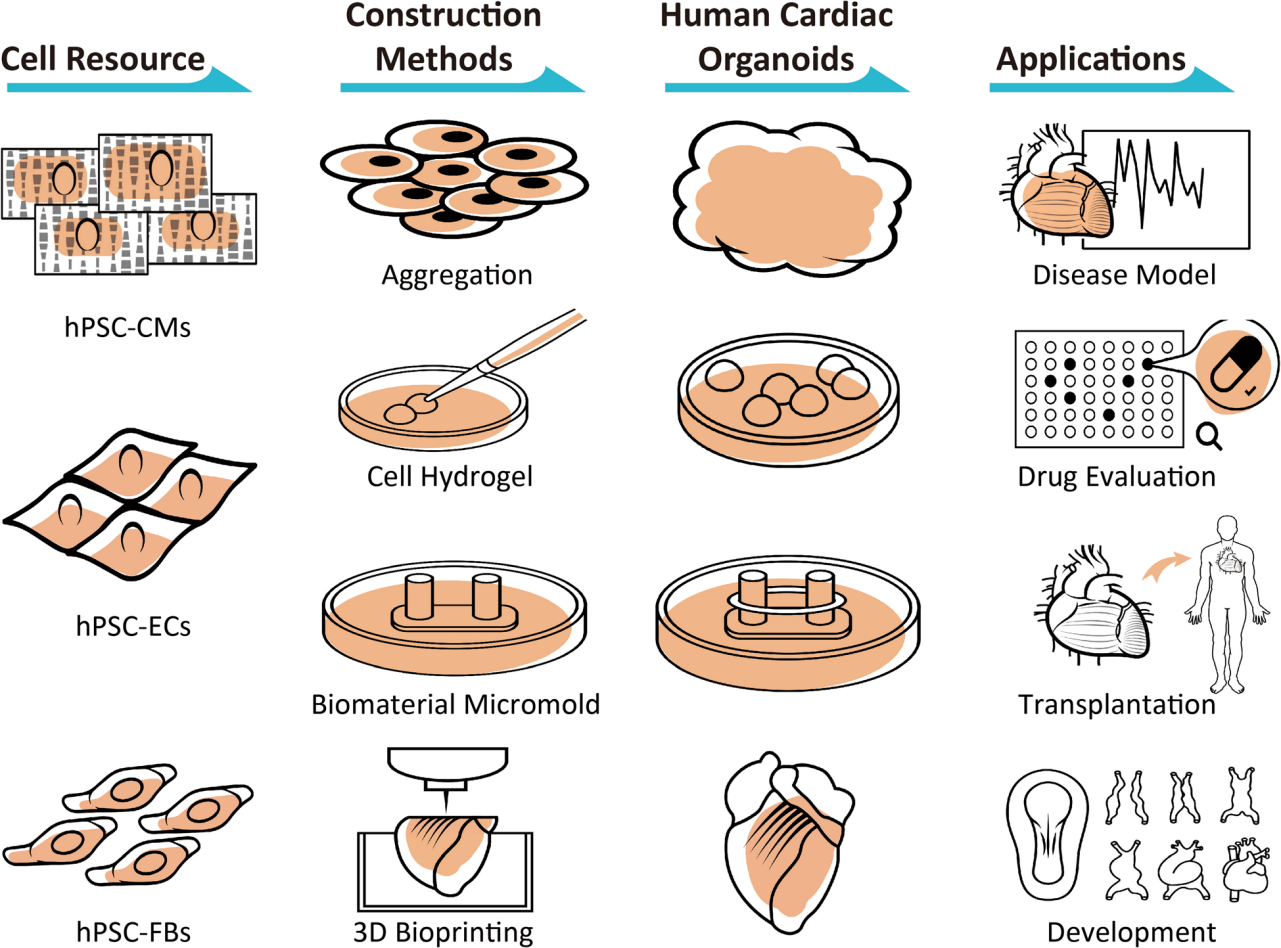
Construction methods and applications of human cardiac organoids. The human cardiac organoid consists of different cardiac lineage cells derived from human pluripotent stem cells. By utilizing diversified bioengineering techniques, cardiac organoids have the ability to simulate some key characteristics of the heart. Cardiac organoids hold promise as tools to model diseases and predict drug responses, and as sources for heart transplantation and cardiac development research. Many other potential applications for preclinical use of this technology await discovery. hPSC-CMs, human pluripotent stem cell-derived cardiomyocytes; hPSC-ECs, human pluripotent stem cell-derived endothelial cells; hPSC-FBs, human pluripotent stem cell-derived fibroblasts

Cardiovascular disease is a leading cause of death worldwide. Human pluripotent stem cell-derived cardiomyocytes (hPSC-CMs) are significant cell sources for disease models, regenerative therapies, and drug discoveries [22]. Scientists have tried to use a variety of strategies, such as gene editing (e.g., mesoderm posterior 1 [23] and delta-like 1 [24]) or adding small molecules (e.g., triiodothyronine [25], dexamethasone [26], and retinoic acid [27]) to improve the efficiency, purity, maturity, and even specification of these cardiomyocytes. However, there are some limitations to two-dimensional (2D) cell culture in terms of cell morphology, viability, differentiation ability, and gene expression. Traditional cell cultures form relatively pure cell lineages that do not reflect the interaction between cells or the communication between cells and the matrix. Similarly, as one of the important means of preclinical experiments, animal models usually are limited by species specificity and heterogeneity [28]. Hence, there are challenges in generalizing effective and mature biological models with cell complexity. As new-generation models, human cardiac organoids (hCOs) retain the biological characteristics and functions of cells in vivo to a great extent. Compared with the traditional 2D cellular model, organoid is more complex, realistic, and accurate. For many years, the term of hCO varied by research groups and biomedical research field and has been used to define different types of in vitro cultures, from tissue explants to organ-on-chip systems [3]. Here, we consider hCO to be a high-fidelity 3D model with specific functions similar to that of the heart. We are mainly concerned with hCO construction methods and hCO applications as disease models (Fig. 2).

## Methods for constructing cardiac organoids

In human cardiac research, scientists are inclined to take advantage of the self-organization property of hPSC-derived cardiac lineage cells to construct hCOs [29–31]. Several attempts have led to successfully constructed cardiac organoids with carefully controlled biocompatible scaffolds designed to emulate organ complexity, including cell hydrogel matrices [32, 33], biomaterial-based micromolds [10, 34], and 3D-printed biomaterials [35, 36].

hCOs are usually prepared by inducing the aggregation of specific cells suspended in a medium or embedded in a 3D gel matrix. Recently, hPSC-derived embryoid bodies (EBs) that are adherent to collagen-conjugated hydrogels have been found to be more effective in formation of myocardium-like tissue with branching vessels than those cultured in a suspended state [32]. This result is due to the collagen-conjugated hydrogel having stiffness similar to that of myocardial tissue, which can promote cell proliferation and codifferentiation into cardiomyocytes. It is simple and easy to facilitate the formation of contractive hCOs and make them convenient to use for translational drug screening research. However, the resulting 3D gel matrix has a consequential limitation: If the matrix is too soft, it is unable to transmit mechanical signals that are insoluble during cardiomyocyte differentiation. Therefore, the difference in gel stiffness results in a great difference in the degree of differentiation.

Surface morphology and electrical field stimulation provide important guidance clues for the organization and contraction of cardiomyocytes in vivo. Recently, Mills et al. fabricated hCOs utilizing a 96-well device with SU-8 photolithography and polydimethylsiloxane (PDMS) casting. This device provides a universal and rapid tool for combining hCOs and preforming drug screening studies. For each hCO, a mixture of hPSC-CMs with type I collagen and matrigel was prepared on ice and then pipetted into the device for high-throughput functional screening. The high-throughput bioengineered hCO platform can be used to investigate the underlying mechanisms of cell cycle arrest [33] and to perform screening for drug discovery [37]. These data exquisitely exemplify that the hCO model provides functional contractile tissues with biological properties similar to those of the native heart and suggest the potential of hCOs as disease treatment platforms.

Histomorphogenesis and organogenesis are the results of biochemical and biophysical cues that lead to spatial patterns of cells during development. To simulate these events in vitro, a patterned micromold has been described for modeling the characteristics of early human cardiac organogenesis. This mold created the necessary geometric constraints to drive the spatial organization of hPSCs and create a beating human cardiac microchamber during differentiation. This model combines organoid concepts with cell micropatterning approaches to generate beating 3D microcavity structure, which are of great significance in terms of experimental systematics and repeatability and allow us to establish a direct link with spatial cellular fate specifications [10, 34]. hCOs with biomaterial-based microchambers are flexible platforms for the design of alternative heart models useful for investigating cardiac pump function and repair mechanisms. However, due to restricted conditions, the beating 3D heart microcavity can be used only to simulate early heart development.

Recently, the world’s first engineered 3D heart, consisting of blood vessels, ventricles and chambers, to be was bio printed with cardiovascular cells derived from human-induced pluripotent stem cells (hiPSCs) [35]. However, due to the lack of a heart conduction system, such as the sinus node, hCOs are incapable of contractile and diastolic function. Shortly after the 3D heart was introduced, a study published in *Science* by Adam Feinberg and his colleagues reported the design of a Bio3D-derived collagen technology (freeform reversible embedding of suspended hydrogels, FRESH) that can successfully capture fully functional components of the human heart at various scales, from capillaries to the entire organ [36]. The key structural, mechanical, and biological properties of natural heart tissue can be constructed through this technology. hCOs using a dual-material printing strategy of myocardial cells and collagen can be thus analyzed for electrophysiological behavior and ventricular contraction associated with arrhythmias. However, many challenges need to be overcome to realize the physiological function of artificial hearts through this model, such as the need to generate the billions of cells necessary for use in the 3D bioprinting of large tissues, the high price of biomaterials, and the requirement for a regulatory process for clinical translation.

Although re-creating a fully functional organ has yet to be achieved, we currently have the ability to recapitulate the structural, mechanical, and biological properties of native tissues by using the aforementioned techniques. The applications of complex hCOs have broad prospects, from observing cardiac disease progression, optimizing drug design, and evaluating drug toxicity (Table 1) [37–48] to providing tools for preclinical testing [49].

**Table 1:** Human cardiac organoids for drug evaluation

Cell types	Production approaches	Analysis	Drug types	References
hiPSC-CMs/hiPSC-ECs/HDFBs	Aggregation	Contraction, immunostaining, histology	Inotropic agent (COA-Cl)	Kitsuka et al. [38]
hiPSC-CMs/hPCFs	Aggregation	LIVE/DEAD staining, ATP activity, beating	Cardiotoxic drugs (astemizole, cisapride, mibefradil, pergoglide, rofecoxib, terodeline, and valdecoxib)	Skardal et al. [39]
hiPSC-CMs/hPCFs	Aggregation	LIVE/DEAD staining, ATP activity, beating	Environmental toxins (glyphosate, lead, mercury, thallium)	Forsythe et al. [40]
hiPSC-CMs/hCFs/hCMECs	Aggregation	Immunostaining, histology, cell viability, real-time qPCR	Cardiotoxic drug (doxorubicin, sunitinib)	Archer et al. [41]
hiPSC-CMs/NHCFs/CEs	HA/gelatin-based hydrogel	LIVE/DEAD staining	Prodrug (capecitabine, ifosfamide)	Rajan et al. [42]
hPSC-CMs/HDFBs	Collagen/matrigel-based hydrogel	Contraction, TEM, immunostaining, teal-time qPCR, RNA sequencing, calcium imaging	Antiarrhythmic drugs (isoprotereno, digoxin, verapamil, nifedipine, disopyramide)	Li et al. [43]
hiPSC-CMs/FBs/HUVECs/HADSCs	Agarose hydrogel micromold	RNA sequencing, transcriptional comparison, force, immunostaining, seahorse, calcium imaging	Heart failure drug (JQ1), cardiotoxic drug (doxorubicin)	Richards et al. [44]
hPSC-CMs/Stromal cells	SU-8 photolithography, PDMS casting	Force, proteomics, immunostaining, RNA sequencing	Pro-proliferative compounds	Mills et al. [37]
hiPSC-CMs	PDMS micropillars	Beating, immunostaining, real-time qPCR, cell viability, calcium imaging	Antidepressant drug (alomipramine)	Yin et al. [45]
hPSC-CMs/HCFs	Polystyrene microwells	Force, TEM, contraction, immunostaining, calcium imaging, RNA sequencing,	Ion channels agents (nifedipine, thapsigargin, ranolazine, verapamil, dofetilide)	Zhao et al. [46]
hiPSC-CMs	Circular cell sheets	Calcium imaging	Antiarrhythmic drugs (quinidine, disopyramide, sotalol)	Shinnawi et al. [47]
hPSC-CMs	Circular casting	Calcium imaging, force, TEM, effective refractory period, real-time qPCR, immunostaining, Western blot	Antiarrhythmic drugs (vernakalant, lidocaine, flecainide, nifedipine carbomylcholin, isoproterenol)	Goldfracht et al. [48]

*CEs* cardiac endothelium cells; *COA-Cl* 2-Cl-C.OXT-A; *HA* hyaluronic acid; *HADSCs* human adipose-derived stem cells; *HCFs* human cardiac fibroblasts; *hCMECs* human cardiac microvascular endothelial cells; *HDFBs* human dermal fibroblasts; *hPCFs* human primary cardiac fibroblasts; *HUVECs* human umbilical vein endothelial cells; *NHCFs* normal human cardiac fibroblasts; *PDMS* polydimethylsiloxane;, *TEM* transmission electron microscopy; *FBs* fibroblasts

## Cardiac organoids for modeling diseases

It is clear that organoids, born in a laboratory dish, can help us better study human physiological functions and pathological mechanisms in ways that traditional cell culture methods and animal models do not allow [21]. Animal models are usually genetically modified or surgically modified to produce a series of symptoms associated with specific diseases [50]. Although animal models have been very useful, the huge differences in gene conservation, metabolism, and longevity between animals and humans have led to bottlenecks in translating animal experimental results into human clinical trials. Organoids are produced by human cells and therefore represent human metabolism and cell turnover rates. To date, considerable progress has been made in the construction of hCOs for cardiovascular modeling. hCOs provide profound benefits for studying of the heart under abnormal conditions [51]. In fact, hCOs may be instrumental in providing the impetus to research additional areas of cardiac physiology, including myocardial ischemic injury, cardiac contractile dysfunction, and abnormal electrophysiological activity [52, 53].

### Myocardial infarction

In a recent study, Voges and colleagues constructed hCOs by using collagen-gel culture of hPSC-CMs in circular molds. They demonstrated that the condition of acute myocardial infarction can be successfully modeled with hCOs that are exposed to local tissue damage using cryoinjury. Moreover, this study is the first to report a model that has the characteristics of fetal-like cardiac tissue. hCOs are consistent with neonatal human heart regeneration and exhibit an endogenous regenerative response with full functional recovery 2 weeks after acute injury. These findings provide insight into the molecular and cellular regulatory mechanisms of immature human heart regenerative capacity [30].

In the latest research, Richards et al. utilized nonadhesive agarose hydrogel molds to make hPSC-CMs and nonmyocyte mixtures used to form hCOs. The hCOs were placed into a hypoxic chamber within an incubator with 10% O_2_ and 1 μM noradrenaline to create an apoptotic gradient, which simulates the environment of myocardial infarction in vitro. By recapitulating the properties of myocardial infarction (especially the pathological metabolic changes, fibrosis, and calcium handling) at the transcriptome, structure, and function levels, this group successfully developed an in vitro organotypic cardiovascular disease model with nongenetic upstream pathological stimuli [44].

Notably, one limitation of the aforementioned hCOs is their inability to include all the cells found in the in vivo heart. Immune/inflammatory cells are known to play important roles in the early response after myocardial infarction and are thought to contribute to the activation of the subsequent fibrotic response [54]. hCOs, due to the lack of inflammatory cells and the use of immature hPSC-CMs, cannot highlight the role of the immune system in myocardial infarction.

### Heart failure

Similarly, using hPSC-CMs and fibroblasts, Tiburcy et al. reported a systematic approach for the formation of hCOs with structural and functional features of the postnatal myocardium. They utilized morphological, functional, and transcriptome analyses to measure the maturation of hCOs, from the shape, systolic twitch forces, and positive force-frequency response to transcriptome profiling. On the basis of the model, they introduced a neurohumoral overstimulation protocol to simulate human heart failure. The response of the hCO model to chronic catecholamine toxicity led to contractile dysfunction, loss of a positive force-frequency response, cardiomyocyte hypertrophy, cardiomyocyte death, adrenergic signal desensitization, and release of biomarkers (N-terminal B-type natriuretic peptide), all of which are distinguishing features of heart failure. Notably, β1-adrenergic receptor and α1-adrenergic receptor blockade by metoprolol and phenoxybenzylamine can partially or completely prevent the pathological phenotype of heart failure. This study provides a proof-of-concept for applying hCO technology to heart failure modeling and shows pharmacological preventive effects in vitro, which may be conducive to the exploitation of new therapeutic strategies [31]. However, the hCOs constructed by this method are very similar to the morphological, structural, and functional properties of the fetal heart at 13 weeks of gestation, and creating a fully adult phenotype remains a major challenge.

### Genetic cardiac diseases

The genetic causes of familial cardiomyopathy, such as hypertrophic cardiomyopathy or dilated cardiomyopathy, have been known for decades. The hiPSC-derived cell models represent many characteristics of human diseases and are useful for studying genetic diseases in the human context. Recently, Yang et al. studied a novel myosin heavy-chain 7 mutation (E848G) identified in familial cardiomyopathy using patient-specific iPSC-CM-derived hCOs to assess contractile function. They generated constructs using a cell suspension containing hiPSC-CMs and human marrow stromal cells and placed them into custom PDMS molds. To measure the tissue construct force, the hCOs were attached to a force transducer and a length controller. Strikingly, there was significantly less alignment in familial cardiomyopathy hCOs than in wide-type hCOs, and there was a significant reduction in systolic function in the E848G-carrying hCOs with minimal impact on diastolic function. Because the contraction of aligned myofibrils was reproduced in 3D system, the findings were more physiologically significant [55].

The introduction of genome-editing techniques has greatly promoted the development of homogenic hPSC lines, which have been widely applied to 2D disease models [56]. In the same way, by combining with genomic editing technology, hCOs can be modified precisely to correct mutations and attain innovative and personalized therapeutic platforms for disease modeling. Clustered regularly interspaced short palindromic repeat (CRISPR)/Cas9 system is an advanced genomic editing technology used for correcting and mitigating disease-causing mutations [57]. In a recent study, Long et al. showed that correction of the cardiomyopathy-associated Duchenne muscular dystrophy (DMD) phenotype requires single-site genomic editing of only 50% of the cardiomyocytes. The authors observed that dystrophin mutations revealed the contractile dysfunction in hiPSC-CM-derived hCOs. By integrating hiPSCs edited with CRISPR/Cas9, they manipulated DMD-hCOs with corrected cells to achieve specific tissue replacement therapy. Importantly, this correction was successfully monitored through an analysis of the force hCO contractions. These data further highlight the usefulness of hCOs as preclinical tools for gene-editing therapy [58]. However, there are some restrictions on the employment of organoids in combination with CRISPR/Cas9 technology. The first limitation involves the efficiency of transfection. Not all cells transfected with the CRISPR/Cas9 component acquire the desired mutation. In addition, the quantity of cells needed to establish organoid systems with genomic mutations is relatively large. Therefore, when conducting CRISPR/Cas9 experiments in organoids, it is crucial to select cells with the desired mutations by the appropriate methods. Solutions to attenuate these drawbacks are being developed [57].

### Arrhythmia

According to existing reports, hCOs have the capacity to produce both spontaneous and induced action potentials, and they have higher conduction velocities than two-dimensional model. Therefore, an hCO is a good disease model to study arrhythmia. In traditional research, hereditary arrhythmia research mainly focuses on the cell characteristics of hiPSC-CMs, that is, action potential characteristics and ion current distribution. hCOs can be used to study more complex electrophysiological phenomena, such as conduction and reentry, in arrhythmogenic syndromes, including short QT syndrome (SQTS). Shinnawi et al. proposed using hCOs with unique hiPSC-derived cardiac cell sheets, which enable the integration of patient-specific hiPSC-CMs, CRISPR/Cas9 genome editing, and organoid technologies to reproduce arrhythmic events. The increased susceptibility of SQTS-hiPSC-derived cardiac cell sheets to the development of re-entrant arrhythmias is consistent with the increased ventricular fibrillation inducibility in SQTS patients. The authors used this hCO model to evaluate the efficacy of new candidate antiarrhythmic agents. The results showed that the application of quinidine and disopyramide, but not sotalol, prolonged action potential duration and suppressed arrhythmogenicity. This study provides evidence of hCOs with the ability to recapitulate the SQTS disease phenotype and new insights into the mechanisms the underlying occurrence, persistence, and treatment of this arrhythmia syndrome [47].

Recently, Goldfracht and colleagues developed hCOs composed of chamber-specific hPSC-CMs. Through supplementation with different concentrations of small molecules (e.g., bone morphogenetic protein 4, activin A, and retinoic acid), hPSCs were induced to differentiate into ventricular or atrial cardiomyocytes. The chamber-specific hPSC-CMs were then pipetted into circular casting molds for condensation and transferred onto a silicon passive stretcher to form chamber-specific, ring-shaped, hCOs. Chamber-specific hCOs display distinct atrial versus ventricular phenotypes including morphology, gene expression, electrophysiological, and contractile properties. More importantly, the majority of the atrial hCOs displayed different types of arrhythmias at baseline, which were terminated by the application of an electrical shock. These results suggest the potential of atrial hCOs as models to study recurrent arrhythmias, such as atrial fibrillation. Consequently, arrhythmic atrial hCOs were used to evaluate the effects of related pharmacological interventions for terminating and preventing the initiation of arrhythmias. The authors confirmed the ability of the antiarrhythmic agents flecainide and vernakalant to terminate re-entrant activity in atrial hCOs [48]. However, the macrostructure of these hCOs is relatively simple and lacks a relative chamber-specific structure. Moreover, the expression of ion channel-related genes and resting membrane potential is not consistent with those of the adult heart.

To date, a cardiac organoid model as a supplement to a preclinical model can be used to simulate pathological processes and heart development effectively and to detect toxicity and side effects of drugs, but it has not yet completely replaced animal models. Cardiac organoids in the laboratory cannot reach physiological shape or size in vivo, nor do they recapitulate multifactorial pathologies by incorporating additional key tissue compartments of native organs (such as the nervous or immune system) [42]. Nevertheless, these studies demonstrate that exploiting hCOs to model cardiac disorders can offer promising prospects for preclinical treatment platforms.

## Outlook and challenges

### Innovative construction methods

**Fig. 3:**
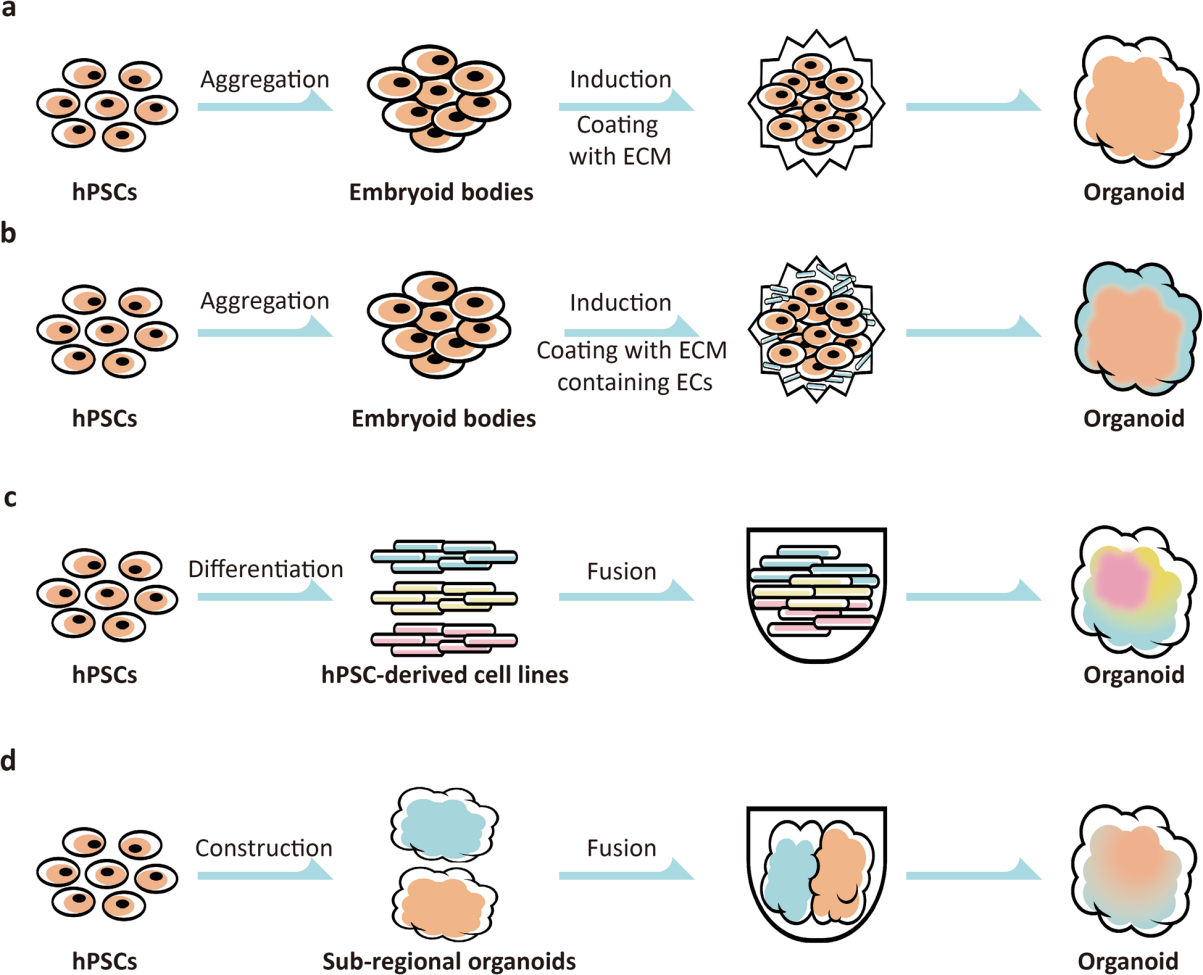
Schematic of self-organizing organoid formation. **a** The typical construction approach of organoids self-assembled from hPSCs. **b** A protocol for the generation of vascularized organoids by coating the organoids with ECM containing ECs. **c** Generation of organoids through the agglutination of multiple types of hPSC-derived cells. d Generation of organoids by fusion of multiple specific subregional organoids. ECM, extracellular matrix; hPSCs, human pluripotent stem cells; ECs, endothelial cells

Currently, due to the deep understanding of extracellular matrix (ECM) biology and the development of suspension culture methods, 3D cellular culture systems play key roles in organoid culture studies [3, 53]. However, despite advances in technology, hCOs are still relatively simplified organ-like tissues, most of which do not contain diverse cellular systems. Therefore, using the construction methods of other organoids for reference will help to build hCOs with multiple cell types and more complete structures. One of the most important problems with hCOs constructed using typical methods is the lack of abundant vascular networks (Fig. 3a). To model the developmental perineural vascular plexus, a strategy was described for the generation of endogenous vascularization by coating the organoids with ECM containing endothelial cells (ECs) (Fig. 3b). This approach seems to be more consistent to the cellular structures involved [59]. In addition to the EB differentiation pathway, Kitsuka et al. cultured cardiac organoids with fibroblasts, hPSC-CMs and hPSC-derived endothelial cells (hPSC-ECs), which have been defined as vascularized [38] (Fig. 3c). In addition, based on existing reports, the fusion of two subregional organoids has also been proposed to form a more complete organ [60] (Fig. 3d). All these innovative coculture methods can better simulate the interaction between multiple cell types and subregions, which will be conducive to constructing specific-chambered hCOs containing ventricle-like and atrial-like structures and functions in the future.

### Extensive application prospects

The advancement of iPSC technology has greatly promoted the rapid development of the biomedical field, and personalized medicine is one of the important outcomes. Personalized medicine, also known as precision medicine, is the clinical practice of medicine according to the individual characteristics of a specific patient [61]. The great potential of personalized medicine lies in moving from a method that is suitable for all to a treatment strategy that is most likely to benefit each individual to improve clinical outcomes and achieve optimal expectations [62]. The unique advantages of iPSCs are their derivation from the patient’s own somatic cells and their genetic match with the person from whom they originated [63]. With a perfectly matched genetic background, the construction of a hCO using patient-specific iPSCs can integrate all genomic loci involved in a drug response, allowing the comparison of an individual patient’s physiological response to drugs. Thus, hCO models provide opportunities to better assess drug efficacy and safety in vitro [64].

Recent studies have further increased the complexity of developmental organoids, focusing on the artificial assembly of multiple organoids or the combination of organoids with cells from different tissue lineages to force aggregation [65]. The organoid-on-a-chip platform is an innovative technology to fabricate 3D organ models, which may bridge the gap between monolayer cell cultures and animal models. The integration of multiorganoids in a culture/analysis chip provides the necessary tools to analyze different parameters that may change under specific conditions [66]. Recently, Yin et al. established a multiorganoids-on-a-chip system derived from hiPSCs, which enable evaluation of cardiac safety of antidepressants with respect to liver metabolism in vitro [45]. This liver-heart organoid-on-a-chip device comprises a partition chamber separated by porous membrane that allows the coculture of 3D human liver organoids in the upper chamber and cardiac organoids in a microcolumn array at the bottom. Based on the combination of stem cell biology and microengineering technology, the proposed hiPSC-derived multiorganoids-on-chip system can reflect the specific functions at the multiorgan level, as well as the complex processes of drug metabolism and reaction.

### Challenges

To truly simulate the structure and function of tissues in vivo, several difficulties related to using organoid technology need to be resolved. First, for instance, the differentiation of organoid cells on a large scale with high efficiency, high quality, and directionality remains a key problem. Second, integrating the frontier advantages of biology, materials science, and bioengineering to construct more precise and complex organs is also urgently needed. A third challenge is caused by the dependence of spontaneous organoid formation on endogenous physiological mechanisms, including various cytokines and signaling pathways between cells and the matrix, as well as those involving multiple cells. It is necessary to precisely coordinate the role of each signaling pathway in the whole process [1, 2, 51, 53, 67]. Finally, nutrients are not evenly distributed by diffusion in the organoids, frequently resulting in a necrotic core and limiting the maximum possible size of the system. Vascularization or long-term culture can promote the development and maturation of organoids, making them larger and more functional. Approaches based on cell biology, genetics, and bioengineering are being used to solve this problem, for instance, introducing endothelial cells or printed vascular structures into organoids [68].

Overall, the solution of these problems will further improve the structural and functional maturity of organoids; provide more accurate models for development, disease, and drug research; and provide original transplant donors for clinical tissue/organ repair.

## Data Availability

Not applicable.

## Abbreviations

2D: Two-dimensional
3D: Three-dimensional
CRISPR: Clustered regularly interspaced short palindromic repeats
DMD: Duchenne muscular dystrophy
EBs: Embryoid bodies
ECs: Endothelial cells
ECM: Extracellular matrix
hCO: Human cardiac organoid
hiPSCs: Human induced pluripotent stem cells
hPSC-CMs: Human pluripotent stem cell-derived cardiomyocytes
PDMS: Polydimethylsiloxane
PSCs: Pluripotent stem cells
SQTS: Short QT syndrome
